# The Role of Oxidative Stress in Atherosclerosis

**DOI:** 10.3390/cells11233843

**Published:** 2022-11-30

**Authors:** Matthew Batty, Martin R. Bennett, Emma Yu

**Affiliations:** Section of Cardiorespiratory Medicine, University of Cambridge, Cambridge CB2 0BB, UK

**Keywords:** oxidative stress, atherosclerosis, mitochondria

## Abstract

Atherosclerosis is a chronic inflammatory disease of the vascular system and is the leading cause of cardiovascular diseases worldwide. Excessive generation of reactive oxygen species (ROS) leads to a state of oxidative stress which is a major risk factor for the development and progression of atherosclerosis. ROS are important for maintaining vascular health through their potent signalling properties. However, ROS also activate pro-atherogenic processes such as inflammation, endothelial dysfunction and altered lipid metabolism. As such, considerable efforts have been made to identify and characterise sources of oxidative stress in blood vessels. Major enzymatic sources of vascular ROS include NADPH oxidases, xanthine oxidase, nitric oxide synthases and mitochondrial electron transport chains. The production of ROS is balanced by ROS-scavenging antioxidant systems which may become dysfunctional in disease, contributing to oxidative stress. Changes in the expression and function of ROS sources and antioxidants have been observed in human atherosclerosis while in vitro and in vivo animal models have provided mechanistic insight into their functions. There is considerable interest in utilising antioxidant molecules to balance vascular oxidative stress, yet clinical trials are yet to demonstrate any atheroprotective effects of these molecules. Here we will review the contribution of ROS and oxidative stress to atherosclerosis and will discuss potential strategies to ameliorate these aspects of the disease.

## 1. Introduction

### Global Burden of Cardiovascular Disease

Cardiovascular disease (CVD) is the most common cause of death globally, with an estimated 32% of all annual deaths being attributed to CVD [1]. The number of cases of CVD has increased from 271 million in 1990 to approximately 523 million in 2019 [2]. These figures are likely to increase further with population growth, ageing, and lifestyle changes associated with economic growth, increasing the burden on healthcare systems worldwide. There is therefore a growing pressure to develop new strategies to manage and reduce cases of CVD.

Underlying the majority of CVD cases is atherosclerosis, a chronic arterial disease characterised by the accumulation of inflammatory cells and lipids in the blood vessel wall. Atherosclerotic plaques may become unstable over time, leading to plaque rupture. This stimulates thrombus formation that can occlude arteries, interrupting the blood supply to vital organs such as the brain and heart. Clinical sequelae ensue, including stroke and myocardial infarction. While research over the last few decades has provided greater insight into the cellular mechanisms that drive atherosclerosis, treating the disease remains a formidable challenge. 

Research has identified that reactive oxygen species (ROS) production is significantly dysregulated during atherosclerosis. ROS production is essential for maintaining vascular health but must be tightly controlled to avoid pro-atherogenic conditions such as inflammation and endothelial dysfunction. Once initiated, these conditions contribute further to oxidative stress due to elevated ROS production and increased failure of cellular antioxidant systems. However, the shared ROS-generating and antioxidant systems found across atherosclerotic cell types offers potential to create therapies that can target multiple aspects of atherosclerosis. We present an overview of the major sources of ROS in atherosclerosis, the consequences of oxidative stress, and strategies that have been used to attenuate vascular oxidative stress. 

## 2. Development of Atherosclerosis

**Figure 1:**
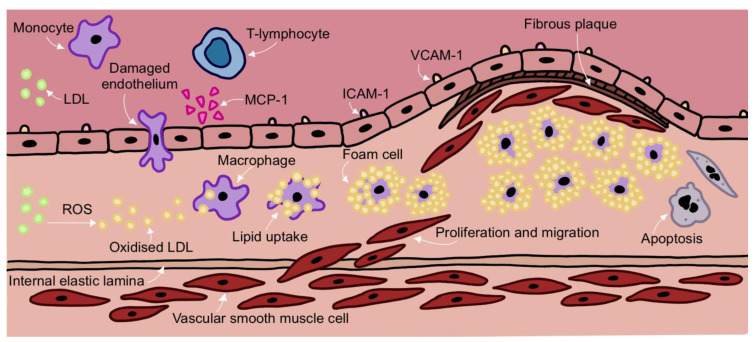
Overview of the Development of Atherosclerosis.

Atherosclerotic plaques often occur at sites of disturbed laminar flow and altered shear stress. This allows the infiltration of lipids and immune cells into the subendothelial space of vessel walls [3]. The trapped LDL particles can become oxidised by ROS and the oxidised LDL (oxLDL) stimulates an inflammatory response. The endothelium becomes activated with endothelial cells expressing cytokines and adhesion molecules. Circulating monocytes are recruited to the site of injury where they attach to the endothelium. The monocytes then migrate into the vessel, differentiate into macrophages and ingest oxLDL. The macrophages develop into lipid-laden foam cells that secrete cytokines and chemokines, stimulating the recruitment of additional monocytes and T-lymphocytes to the subendothelial space. The inflammatory cell/lipid deposit forms the initial fatty streak that can develop into the more complex atherosclerotic plaque [4] (Figure 1). 

Vascular smooth muscle cells (VSMCs) usually reside in the arterial medial layer, although humans also have VSMCs in their intimas. However, the release of mitogens, inflammatory cytokines, and chemoattractants, including PDGF and TNF-α, recruits VSMCs to the intima. VSMCs switch from a contractile to synthetic phenotype, secreting extracellular matrix (ECM) that forms a protective fibrous cap around the core of the atheroma. However, fibrous cap integrity can become compromised resulting in cap thinning and potential rupture. When the plaque ruptures, the thrombogenic core is exposed stimulating platelet aggregation. The subsequent thrombus may occlude the artery resulting in ischaemia and infarction of the downstream vascular territory.

LDL becomes trapped in the subendothelial space. In response, endothelial cells secrete chemokines such as MCP-1 which attract circulating monocytes to the vascular endothelium. Other classes of leukocytes such as T-lymphocytes are recruited to the endothelium and contribute to sustained local inflammation. Endothelial cells also upregulate the expression of adhesion molecules such as ICAM-1 and VCAM-1 which allow monocytes to adhere to the endothelium and infiltrate into the subendothelial space. Monocytes differentiate into macrophages in response to the high local concentrations of cytokines and mitogens in the inflamed vessel wall. Inflammation contributes to a significant generation of ROS which oxidise LDL particles trapped in the subendothelial space. Macrophages engulf oxidised LDL and differentiate into lipid-laden foam cells which lose their migratory capacity and accumulate to form the fatty core of the plaque. Meanwhile, inflammatory conditions stimulate vascular smooth muscle cells (VSMCs) to migrate from the vessel wall media layer to the intima through the internal elastic lamina. VSMCs contribute to the production of extracellular matrix components such as collagen, fibronectin and elastin which form a protective fibrous cap around the fatty core of the plaque. Foam cells and VSMCs may undergo apoptosis and the presence of dead cells contributes to necrotic core formation. Low density lipoprotein (LDL), reactive oxygen species (ROS), monocyte chemoattractant protein (MCP-1), intracellular adhesion molecule 1 (ICAM-1), vascular cell adhesion molecule (VCAM-1). 

## 3. Overview of ROS

ROS are oxygen-containing species with high reactive properties that are generated by the reduction of oxygen [5]. The group includes free radical species such as superoxide (O_2_·^−^) and hydroxyl species (HO·) and non-free radicals such as hydrogen peroxide (H_2_O_2_). Once formed, these can produce more complex species such as peroxynitrite (ONOO^-^), hypochlorous acid (HOCl) and lipid peroxyl radicals. Peroxynitrite may react further with carbon dioxide to form highly reactive nitrosperoxycarbonate. Physiological levels of ROS are necessary to maintain the signalling pathways that control cellular processes such as inflammation, differentiation, proliferation and apoptosis. However, dysregulated ROS production can lead to the overstimulation of these pathways which can promote ROS-associated disease phenotypes as seen in type II diabetes (TIID), cancer and atherosclerosis [6]. Importantly, elevated ROS production is a significant risk factor for further ROS generation, a phenomenon known as ROS-induced ROS release, which promotes further disease progression [7]. 

## 4. Sources of ROS in Atherosclerosis

**Figure 2:**
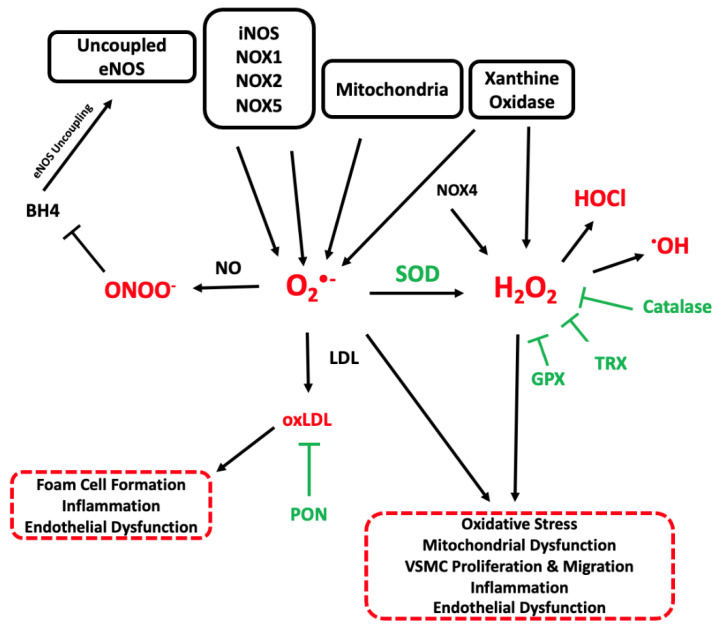
Overview of Vascular Sources and Functions of ROS in Atherosclerosis.

ROS can be generated by several enzyme systems found throughout the vascular system. Significant sources of vascular ROS include the mitochondrial electron transport chain, NADPH oxidases (NOX), xanthine oxidase and endothelial nitric oxide synthase (eNOS) (Figure 2). Broadly, these enzymes catalyse the reduction of oxygen by transferring electrons from their respective substrates to oxygen molecules. Basal levels of ROS are required for cellular homeostasis and signalling. Elevated ROS can be beneficial, most notably in the macrophage oxidative burst that is required for pathogen killing. However, when ROS are in excess, which may be due to increased ROS generation and/or decreased antioxidant capacity, oxidative stress results. 

Superoxide (O_2_^·-^) can be produced by a variety of enzymatic sources including uncoupled endothelial nitric oxide synthase (eNOS), inducible nitric oxide synthase (iNOS), NADPH oxidases (NOX1, NOX2 and NOX5) and by mitochondria. Superoxide anions may react with nitric oxide (NO) from eNOS and iNOS to form peroxynitrite radicals (ONOO^-^). Peroxynitrite scavenges the essential eNOS cofactor, tetrahydrobiopterin (BH4) which leads to eNOS uncoupling and further superoxide production. Superoxide ions may also be dismutated into less reactive hydrogen peroxide (H_2_O_2_) molecules by superoxide dismutase (SOD) enzymes expressed in the extracellular matrix and mitochondria. Hydrogen peroxide may also be produced directly by xanthine oxidase and NOX4 and is cleared by catalase, glutathione peroxidase (GPX) and the thioredoxin (TRX) system. Furthermore, hydrogen peroxide can be converted to hypochlorous acid (HOCl) or hydroxyl (·OH) species. Reactive oxygen species (ROS) can react with low density lipoproteins (LDL) to form oxidised LDL (oxLDL), a key pathogenic step in atherosclerosis which promotes foam cell formation, endothelial dysfunction and additional inflammation. Paraoxonase (PON) enzymes can bind to high density lipoprotein (HDL) and reduce LDL and HDL oxidation.

### 4.1. Mitochondrial ROS

Mitochondria are organelles that have four key components: an outer lipid bilayer membrane, an intermembrane space, an inner membrane and an internal matrix.

Mitochondria are major cellular energy generators found in all nucleated human cell types. In addition to their primary role of generating ATP by oxidative phosphorylation (OXPHOS) they play vital roles in calcium and iron homeostasis, and regulating critical processes including ROS generation, inflammation and apoptosis [8]. However, mitochondrial dysfunction can develop and is found in a broad range of diseases including cancer, neurodegeneration and atherosclerosis.

Many signalling pathways use mitochondrial ROS (mtROS) as cellular messengers. mtROS are therefore essential for regulating cellular responses such as gene expression [9], signal transduction [10] and responses to stress [11]. The two major forms of mtROS are superoxide and hydrogen peroxide. During OXPHOS, electrons flowing down the electron transport chain (ETC) can be transferred from complex IV onto molecular oxygen to form water. However, mtROS are formed when electrons ‘spill’ onto oxygen from mitochondrial proteins located earlier on in the ETC.

Complex I is considered the major site of mtROS production but they may also be derived from Complexes II or III [12]. Complex I can generate ROS during both forward electron transfer (FET) or reverse (RET) [13]. During FET, ubiquinone (CoQ) receives electrons from multiple sources including complexes I and II. However, electrons may leak during NADH oxidation at the flavin mononucleotide (FMN) site of Complex I to produce mtROS [14]. ROS can also be formed at the CoQ binding site in CI. mtROS generated during FET serve as redox messengers under resting conditions. However, RET occurs at Complex I when the proton motive force (Δp) across the mitochondrial inner membrane is high and the ubiquinone electron carrier pool is highly reduced. Ubiquinol (reduced CoQ) transfers electrons to complex I with the reduction of NAD^+^ to NADH. However, some of these electrons will leak and reduce oxygen to form superoxide. RET may occur in response to cellular stress or mitochondrial damage during which there is diminished OXPHOS capacity.

### 4.2. NADPH Oxidase

NADPH oxidase (NOX) expression is associated with the severity of atherosclerosis [15]. In mammals there are 7 isoforms of NADPH oxidases: NOX1-5, DUO1 and DUOX2 with NOX1, NOX2, NOX4 and NOX5 being the major forms expressed in the human vasculature. NOX expression has been detected in macrophages, VSMCs, endothelial cells and fibroblasts [16]. NAPH oxidases are primarily expressed by macrophages in response to pathogen invasion but under resting conditions are only expressed at low levels in other cell types. NADPH oxidases catalyse the reduction of molecular oxygen (O_2_) by NADPH, producing the reactive oxygen species superoxide. NOX-derived superoxide can then promote further ROS generation by increasing xanthine oxidase ROS production [17]. Excess superoxide scavenges nitric oxide, generating peroxynitrite [18]. In the case of NOX4, hydrogen peroxide can be directly produced instead of superoxide therefore NOX4 activity does not lead to NO scavenging.

#### NADPH Oxidase in Atherosclerosis

Different isoforms of NADPH oxidase have been implicated in several aspects of atherosclerosis. NOX1 expression is increased in the plaques of patients with cardiovascular events or diabetes [19], and deleting NOX1 reduces lesion area in both apolipoprotein E deficient (ApoE^−/−^) mice and in ApoE^−/−^ mice after induction of diabetes [20,21]. These studies also showed that deletion of NOX1 is associated with decreased ROS generation, leukocyte adhesion and macrophage infiltration. 

Similarly, macrophage and endothelial NOX2 expression is increased in the aortas of ApoE^−/−^ mice and is associated with plaque development and elevated ROS [22]. Atherosclerotic plaques often develop at sites of altered shear stress, which may also promote changes in NOX2 expression. Indeed, oscillatory blood flow-induced shear stress increases the expression of gp91^phox^ (NOX2) in bovine aortic endothelial cells (BAECs) [23], with a corresponding increase in superoxide production and LDL oxidation. Conversely, NOX2 expression was reduced under conditions of pulsatile flow, leading to reduced endothelial superoxide production and LDL oxidation [23]. 

In contrast to NOX1 and NOX2, NOX4 mRNA is decreased in human and mouse plaques. Unlike other NADPH oxidases, NOX4 produces hydrogen peroxide rather than superoxide [24]. Interestingly, deleting NOX4 in ApoE^−/−^ mice reduced hydrogen peroxide formation but increased plaque area [19,25]. Furthermore, plaque NOX4 mRNA and hydrogen peroxide levels are decreased in plaques from patients with symptomatic carotid artery stenosis and may be linked to plaque stability [26]. Taken together this suggests that NOX4, an enzymatic source of ROS, can protect against atherosclerosis. The protective properties of NOX4 may relate to its ability to generate hydrogen peroxide rather than superoxide, as hydrogen peroxide can then stimulate endothelial NO production [27].

The NOX5 gene is absent from rodent species and this has hampered our understanding of its role in atherosclerosis. A recent study using knock-in mice expressing human NOX5 in endothelial cells showed that NOX5 does not promote atherosclerosis [28], although deletion of NOX5 in New Zealand White rabbits significantly increased plaque development in the thoracic aorta, suggesting a protective role of NOX5 [29]. However, the role of NOX5 in human atherosclerosis remains unclear. NOX5 in humans is a major source of ROS and contributes to both endothelial dysfunction and hypertension so may contribute to atherosclerosis [30].

It is evident that the expression of NADPH oxidases and effects on vascular redox status have a clear role in regulating atherosclerosis. Further investigation is required to fully characterise expression patterns and activity levels of NADPH isoforms to determine how they may promote or protect against disease.

### 4.3. Xanthine Oxidoreductase

Xanthine oxidase (XO) is one of two interconvertible forms of xanthine oxidoreductase. XO catalyses the oxidation of hypoxanthine to uric acid in a reaction that produces superoxide and hydrogen peroxide from oxygen. The constitutively expressed xanthine dehydrogenase (XDH) form uses NAD+ as an electron accepter and causes superoxide production under hypoxic conditions. 

There is increasing evidence that XO is involved in the pathogenesis of atherosclerosis [31]. XO expression is significantly higher in plaques from symptomatic atherosclerosis patients compared with asymptomatic plaque [32]. More specifically, plaque macrophages from these patients exhibited significant XO upregulation. Increased XO expression may be a result of oscillatory shear stress, as oscillatory shear stress increases xanthine oxidase:xanthine dehydrogenase ratio in endothelial cells, increasing superoxide production [17]. 

The product of XO reactions, uric acid, is also associated with atherosclerosis. Increased serum uric acid levels are associated with both vulnerable plaque morphology and coronary heart disease mortality [33,34]. Uric acid may promote atherosclerosis through inducing mitochondrial dysfunction, ROS production and inflammasome activation [34]. However, a direct role of uric acid in atherogenesis has yet to be proven. We also note that uric acid itself is characterised as an antioxidant through its peroxynitrite scavenging capacity [35]. 

Pre-clinical data has suggested that targeting xanthine oxidase may be useful in treating atherosclerosis. Inhibiting xanthine oxidase with allopurinol reduces the transformation of macrophages to foam cells after lipid loading [36]. Furthermore, treating ApoE^−/−^ mice with either allopurinol or febuxostat (a non-purine XO inhibitor) reduces plaque formation [36,37]. Despite this promising data, the benefit of allopurinol in humans is yet to be established. In a large case-control study allopurinol use in older patients with hypertension was associated with decreased stroke risk and cardiovascular events [38]. However, the recently published ALL-HEART study, a multi-centre randomised controlled trial, showed no effect of allopurinol on cardiovascular outcomes in patients with ischaemic heart disease [39]. Research into XO inhibitors continues and recent in-silico studies have suggested other molecules, such as amentoflavone, may also be effective XO inhibitors [40].

### 4.4. Uncoupled Nitric Oxide Synthase

Endothelial nitric oxide synthase (eNOS) is the major source of vascular nitric oxide (NO) which acts as a powerful vasodilator through its relaxing effects on VSMCs. Further effects of NO include reduced leukocyte attachment to endothelial cells, reduced VSMC proliferation and inhibition of platelet aggregation [41]. 

However, eNOS can become uncoupled in conditions of oxidative stress. In atherosclerosis oxidised LDL stimulates NAPDH oxidase, increasing superoxide production. Superoxide reacts with NO to form peroxynitrite, which oxidises the essential eNOS cofactor tetrahydrobiopterin (BH4) leading to BH4 deficiency [42]. eNOS therefore becomes uncoupled and generates superoxide, promoting further uncoupling. 

The role of eNOS in atherosclerosis is yet to be fully determined with conflicting data from animal models. In one study, overexpression of eNOS in ApoE^−/−^ mice was associated with reduced lesion size [43] whilst another study found eNOS overexpression increased atherosclerosis [44]. Similarly lack of eNOS has been associated with both increased atherosclerosis [45] or reduced lesion burden [46]. The role of eNOS in atherosclerosis is therefore unclear and may relate to its ability to produce both anti-atherogenic NO and pro-atherogenic superoxide radicals depending on disease stage and oxidative status of the vascular endothelium.

### 4.5. iNOS in Atherosclerosis

Under normal conditions inducible NOS (iNOS) is only expressed at low levels with basal levels of NO produced by constitutively expressed isoforms such as eNOS. iNOS is therefore not considered to be an integral part of maintaining vascular health. Instead, iNOS expression is associated with injury and is an important marker and driver of inflammation [47]. Expression of iNOS predominantly leads to the production of NO from L-arginine. However, similar to eNOS, BH4 deficiency promotes superoxide rather than NO generation [48,49].

Cytokines released in response to lesion formation cause high levels of iNOS expression in VSMCs [50] and macrophages [51]. Cytokines such as TNF-α, IFN-γ and IL-1 bind to cell surface receptors and stimulate the transcription factors STAT1a and NFκB to promote iNOS expression. iNOS generates significant amounts of NO (up to a 1000-fold increase compared to physiological levels) [52] which can be damaging to tissues as NO can react with superoxide to generate peroxynitrite.

Elevated iNOS expression has been observed in human atherosclerotic plaques and is associated with features of plaque instability, such as symptoms of unstable angina or the presence of thrombus formation [53]. iNOS deficiency in ApoE^−/−^ mice reduces lesion size without altering plasma cholesterol levels [54]. Additionally, depletion of iNOS in ApoE^−/−^ mice reduces plasma lipoperoxides, indicating reduced oxidative stress which may have inhibited plaque growth [55]. 

## 5. Antioxidant Systems in Atherosclerosis

In addition to changes in the expression and activity of ROS-producing enzymes, the failure or overwhelming of endogenous antioxidant systems is a common feature in atherosclerosis that promotes oxidative stress. The major antioxidant systems found in the vasculature include superoxide dismutase (SOD), catalase, thioredoxins, paraoxonases, glutathione peroxidase (GPX) and mitochondrial uncoupling proteins (UCP) [56]. Under physiological conditions, ROS production is relatively low and excess ROS are adequately scavenged by antioxidant systems. However, during atherosclerosis, ROS production may increase significantly and antioxidant systems may become downregulated. This results in imbalanced ROS production and oxidative stress. 

SOD enzymes convert superoxide radicals to less reactive hydrogen peroxide which can then be degraded by catalase and glutathione peroxidase. Patients with coronary artery disease demonstrate elevated SOD expression during the initial stages of disease. However, SOD later becomes downregulated as the disease progresses [57]. Similarly the expression of manganese SOD (MnSOD), which is localised to the mitochondrial matrix, is downregulated in peripheral blood mononuclear cells from coronary artery disease patients [58]. Loss of manganese superoxide dismutase activity in ApoE^−/−^ mice promotes atherosclerosis [59].

SOD3 is the important antioxidant enzyme in the extracellular matrix. NO upregulates SOD3 expression in human VSMCs [60]. However, SOD3 expression may be decreased in atherosclerosis due to reductions in NO bioavailability [61]. The effect of SOD-3 on atherogenesis is unclear; after one month of high fat feeding SOD-3 deletion led to a slight decrease in plaque area but by three months no differences were observed [62]. The effects of SOD on atherosclerosis may depend on the redox state of the vasculature. SOD can be protective by limiting the damage induced by superoxide. However, if there is insufficient GPX or catalase, then SOD-produced hydrogen peroxide can increase oxidative stress. 

Glutathione peroxidase reduces hydrogen peroxide to water and oxygen, and lipid hydroperoxides to their corresponding alcohols. Liposomal glutathione administration to ApoE^−/−^ mice reduces the formation of lipid peroxides, reduces macrophage cholesterol uptake and decreases lesion area [63]. Furthermore, ApoE^−/−^ mice deficient for glutathione peroxidase show increased atherosclerosis [64]. This may be due to increased oxLDL induced- foam cell generation and macrophage proliferation via the p44/42 MAPK (ERK1/2) signalling pathway [65].

Catalase, a peroxisomal enzyme, also converts hydrogen peroxide to oxygen and water. Catalase expression is upregulated in response to the presence of lipid peroxides in vitro [66]. Overexpression of catalase slows the progression of atherosclerosis in ApoE^−/−^ mice [67]. 

Paraoxonases (PON) are a family of three lactone hydrolysing enzymes (PON1, PON2, PON3) with important roles in regulating oxidative stress and inflammation. PON1 is the best studied paraoxonase and is found in HDL particles where it can hydrolyse peroxide phospholipids to prevent the oxidation of both HDL and LDL particles [68]. Overexpression of the human *PON1* in ApoE^−/−^ mice and in PON1-transgenic B6 mice fed a high fat diet significantly reduced lesion size without affecting total plasma cholesterol levels [69]. This may reflect the ability of PON1 to stimulate cellular cholesterol efflux through ABCA1, PPARγ and LXRα upregulation, as shown in *PON1* expressing J774 macrophages [70]. Furthermore, PON2 is expressed in the vessel walls and is found in mitochondria where it is associated with Complex III and reduces superoxide production. PON2/ApoE^−/−^ double knockout mice show increased atherosclerosis with elevated mitochondrial oxidative stress [71]. PON3 is found in both mitochondria and associated with HDL particles. In patients with subclinical atherosclerosis PON3 is depleted from HDL particles [72].

The thioredoxin system (TRX) is an important disulfide-reductase antioxidant system which utilises the reducing power of NADPH to remove hydrogen peroxide and maintain the redox status of the cell. It is composed of thioredoxin reductase-1 (TRXR1), thioredoxin-1 (TRX1) and thioredoxin-interacting protein (TXNIP). TRXR1 transfers electrons from NADPH to TRX1 to maintain a pool of reduced TRX1 and thus the reducing cytosolic environment needed for protein assembly. This can be attenuated by TXNIP which binds to the catalytic centre of reduced TRX1 to inhibit its function [73]. TRX1 is upregulated in human atherosclerotic plaques and is expressed in foam cells [74]. Plasma levels of TRX1 are also increased in patients with chronic heart failure [75]. TRX1 expression is therefore linked to atherosclerosis and reduces lesion formation via NLRP3 inflammasome inhibition in ApoE^−/−^ mice [76].

Mitochondrial uncoupling proteins have a critical role in atherosclerosis through their ability to regulate the proton gradient across the inner mitochondrial membrane and thus govern ROS release [77]. Expression of the rs5977238 *UCP5* single nucleotide polymorphism is associated with a decreased risk of carotid plaque formation [78]. A protective role of UCP2 in atherosclerosis has also been suggested as mice transplanted with bone marrow from UCP2^-/-^ mice demonstrated elevated oxidative stress and increased lesion size [79].

An important regulator of antioxidant systems is the transcription factor nuclear factor E2 related factor 2 (NRF2). In response to stress, NRF2 increases the expression of a number of cytoprotective genes including the anti-oxidant enzymes SOD, catalase and glutathione peroxidase [80]. Under basal conditions NRF2 activity is regulated by the Keap1/Cullin3 ubiquitin ligase complex. Ubiquitination of NRF2 targets it for degradation by the 26S proteasome. However, under stress Keap1 binding to NRF2 is reduced allowing NRF2 to translocate to the nucleus and activate the anti-oxidant response [81]. NRF2 might therefore be expected to have an atheroprotective role. Indeed, NRF2 expression protects human aortic endothelial cells from oxidant-mediated cytotoxicity and NRF2 activation at atheroprotected sites is associated with decreased VCAM-1 expression [82,83]. Furthermore, myeloid deletion of NRF2 reduces catalase expression, and increases inflammation and atherosclerosis [84]. However, NRF2 also has effects on lipids that might promote atherosclerosis. Whole body NRF2 knock out reduces both antioxidant gene expression but also atherosclerosis. This may be due to decreased total plasma cholesterol levels, decreased expression of the CD36 scavenger receptor and effects on cholesterol crystal-induced inflammasome activation [85,86]. 

## 6. The Effects of ROS in Atherosclerosis

ROS have multiple effects on the vasculature that promote atherosclerosis. These include oxidative modification of lipids and DNA, endothelial dysfunction and inflammation. ROS can also affect the stability of the plaque fibrous cap. 

### 6.1. Lipid Oxidation

During atherogenesis LDL accumulates in the arterial wall, particularly at sites with disturbed flow. LDL then undergoes oxidative modification that can be mediated by ROS generated from NADPH oxidase or uncoupled eNOS. Oxidised LDL is pro-atherogenic, impairing endothelial production of NO and inducing the expression of leukocyte adhesion molecules [87]. Decreasing LDL oxidation through deletion of lipoxygenases reduces atherosclerosis [88] whilst increased LDL oxidation increases lesional area [89]. 

Mitochondrial lipids are also a major target for ROS-induced oxidative damage. Cardiolipin makes up to 20% of the lipid content of mitochondrial membranes and is prone to oxidative damage due to its high number of unsaturated fatty acids. Cardiolipin content is reduced and lipid peroxidation increases in mitochondria isolated from rat liver after ischaemia reperfusion injury [90]. This can be explained by the burst of mtROS produced at Complex I that occurs shortly after the oxidation of accumulated succinate upon restoration of ETC function after ischemia [91]. Oxidised cardiolipin levels and levels of endogenous anti-cardiolipin antibodies are increased in atherosclerotic plaques [92]. Furthermore, oxidised cardiolipin is a potent proinflammatory signal which induces the expression of leukotriene and 5-lipoxygenase by leukocytes and the expression of adhesion molecules such as ICAM-1 and V-CAM1 by endothelial cells [93].

### 6.2. DNA Oxidation

DNA is susceptible to oxidative damage by ROS and resultant lesions include single stranded breaks, double stranded breaks, adducts and deletions [94]. Patients with coronary artery disease show increased chromosomal damage in peripheral lymphocytes [95]. Levels of 7,8-dihydro-8-oxo-2’-deoxyguanosine (8-oxo-dG), a marker of DNA oxidative damage, are also increased in plaque macrophages, smooth muscle cells and endothelial cells [96]. Nuclear DNA damage is not merely a bystander in atherosclerosis but contributes to disease development. ApoE^−/−^ mice haploinsufficient for the DNA repair protein kinase ATM (ataxia telangiectasia mutated) show increased nuclear DNA damage and accelerated atherosclerosis [94]. Inhibiting oxidative DNA damage through improving base excision repair markedly reduces plaque size [97]. 

In the mitochondria the ROS-producing sites of Complex I are located on the matrix face of the inner membrane, such that superoxide will diffuse into the matrix. As mitochondrial DNA (mtDNA) is located in the matrix and lacks protective histones it is at significant risk of oxidative damage by mtROS. Indeed, mtDNA damage is observed in arteries and blood cells of patients with atherosclerosis [59,98]. Furthermore mtDNA damage promotes atherosclerosis in ApoE^−/−^ mice with defective mtDNA polymerase proof-reading activity and is associated with features of plaque vulnerability [98]. 

### 6.3. Endothelial Dysfunction

The vascular endothelium is a monolayer of endothelial cells that line the blood vessel wall and acts as a boundary between circulating blood and tissues. The vascular endothelium helps to modulate vascular tone, control blood flow and is involved in the regulation of the immune response. A dysfunctional endothelium, characterised by endothelial leakage, elevated ROS production, proinflammatory cytokine secretion, increased expression of surface adhesion markers and decreased NO production is a critical component of atherogenesis [99]. This is because it potentiates the risk for circulating LDL to become trapped in the subendothelial space and be subject to oxidative modifications.

The endothelium is both a source and target of ROS. Endothelial cells are a major source of NO due to the constitutive expression of eNOS. However, endothelial cells are also important sources of superoxide and peroxynitrite due to eNOS uncoupling in response to BH4 depletion. Indeed, in ApoE^−/−^ mice eNOS deficiency reduces superoxide production, indicating that eNOS uncoupling occurs during atherosclerosis [100]. The increase in ROS can then be amplified. Chronic exposure to superoxide stimulates mtROS production in mouse vascular endothelial cells which results in decreased proliferation [101]. 

Oxidative stress promotes the proinflammatory endothelium that is necessary for atherosclerosis. Hydrogen peroxide supplementation increases granule membrane protein-140 expression which facilitates neutrophil binding to the surface of endothelial cells [102]. Oxidative stress also induces NFκB which promotes the expression of adhesion molecules such as VCAM-1, ICAM-1 and E-selectin, and cytokines such as TNF-α [103]. In turn, TNF-α induces mtROS production, NADPH oxidase activity and iNOS expression in endothelial cells [103,104]. Endothelial oxidative stress therefore promotes inflammation which can drive further ROS production. 

Oxidised LDL upregulates the expression of oxidised low-density lipoprotein receptor-1 (LOX-1) in endothelial cells [105]. Upregulation of LOX-1 promotes endothelial dysfunction by triggering apoptosis and inflammation [105]. LOX-1 also mediates downregulation of eNOS by oxLDL [106]. Importantly, endothelial LOX-1 expression is associated with atherosclerosis progression. Endothelial-specific *LOX-1*-expressing ApoE^−/−^ mice displayed elevated ROS production, plaque formation, eNOS uncoupling and macrophage infiltration [107]. 

### 6.4. Inflammation

**Figure 3:**
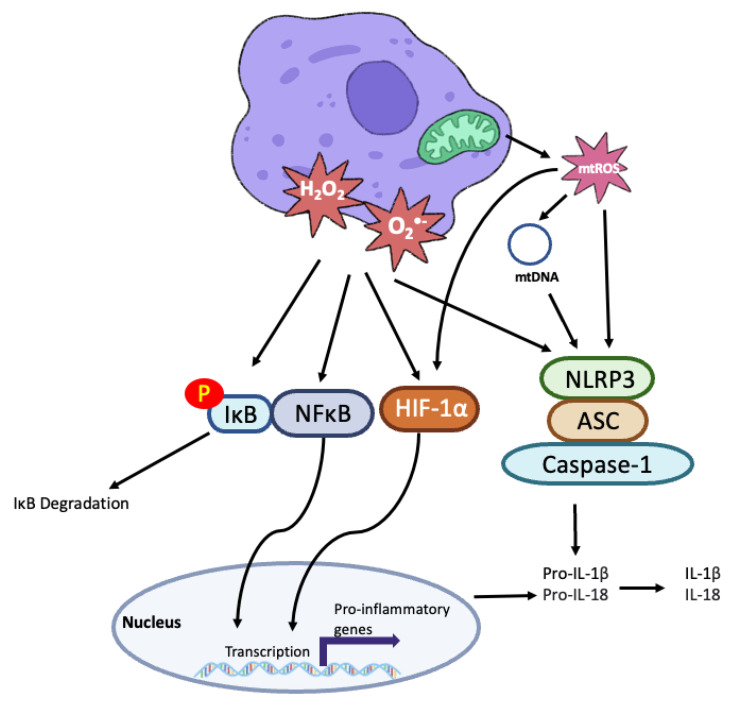
Overview of ROS in Vascular Inflammation.

ROS acts via multiple targets including NFκB, HIF-1α and the NLRP3 inflammasome to promote inflammation (Figure 3), a key process in atherogenesis. NFκB is a transcription factor that regulates inflammatory cytokine expression therefore its activation must be tightly controlled. NFκB is usually held in the cytoplasm by inhibitory IKB proteins. However, IκB phosphorylation by the IKK complex can allow NFκB to translocate to the nucleus and induce the transcription of pro-inflammatory genes including TNFα, IL-1β, IL-18 [108]. In HUVECS treatment with hydrogen peroxide activates NFκB via tyrosine phosphorylation of IκB [109,110]. NFκB can also be activated by superoxide produced by NADPH oxidases. Lipopolysaccharide (LPS), a component of bacterial cells walls, binds to TLR4 which associates with NOX4 to induce ROS generation and NFκB activation [111]. Of importance in atherogenesis, endothelial NOX can be activated under conditions of low shear stress to increase NFκB signalling [112,113]. Bone morphogenic protein 4 induces NOX1 mRNA expression and subsequent hydrogen peroxide and superoxide production [113]. 

ROS can also act via HIF-1α to promote inflammatory cytokine expression. Monocytes undergo a profound metabolic shift when stimulated by LPS to differentiate into M1 macrophages. M1 macrophages primarily rely on glycolysis for ATP generation, leading to increased glucose uptake and decreased mitochondrial OXPHOS. This shift to glycolysis is essential for the expression of some proinflammatory genes such as IL-1β [114]. Reduced dependence on OXPHOS leads to an accumulation of succinate. Succinate may then be oxidised by succinate dehydrogenase to drive a burst of ROS production which stabilises HIF-1α to promote IL-1β expression [115]. 

ROS also promote inflammation via the NLRP3 inflammasome. Activation of the NLRP3 inflammasome is required for the generation of mature IL-1β and IL-18. The NLRP3 inflammasome is composed of NLRP3, the adapter protein ASC and caspase 1, which cleaves pro-IL-1β and pro-IL-18 to their mature forms. Xanthine oxidase-derived ROS have been shown to be a critical regulator of NLRP3 inflammasome activation in macrophages [116]. Activation of NLRP3 by ROS leads to IL-1β and IL-18 expression but inhibiting xanthine oxidase with febuxostat can attenuate this [37]. 

Mitochondrial oxidative stress is also an important regulator of NLRP3 inflammasome activation. Increasing mitochondrial ROS, through inhibition of ETC enzymes or inhibition of autophagy/mitophagy triggers NLRP3 inflammasome activation [117]. mtROS can oxidise mtDNA, which acts as a damage associated molecular pattern to activate the NLRP3 inflammasome [118]. ROS can therefore promote both inflammatory cytokine transcription and post translational modification to increase inflammation. 

Increased Il-1β expression is particularly important in atherosclerosis. Il-1β increases the expression of adhesion molecules and chemokines, such as ICAM-1, VCAM-1 and MCP-1, which recruit leukocytes and mononuclear phagocytes at the initial stages of atherosclerosis [119,120]. Il-1β also acts a powerful mitogen for human SMC and promotes further inflammation through auto-induction and stimulating IL-6 production [121,122]. The importance of Il-1β in atherosclerosis was confirmed in the landmark CANTOS trial. Treatment with canakinumab, a monoclonal antibody that selectively neutralizes Il-1β, reduced the primary endpoint of non fatal myocardial infarction, non fatal stroke or cardiovascular death [123].

During periods of oxidative stress, cellular superoxide (O_2_^·-^) and hydrogen peroxide (H_2_O_2_) are increased alongside a burst of mitochondrial reactive oxygen species (mtROS) production. These reactive oxygen species (ROS) cause the phosphorylation (P) of IκB, leading to its degradation and the release of NFκB. NFκB can therefore translocate to the nucleus where it stimulates the transcription of pro-inflammatory genes such as IL-1β and IL-18. ROS also stabilize HIF-1α, leading to HIF-1α-associated pro-inflammatory gene expression. Activation of the NLRP3 inflammasome, consisting of NLRP3, the ASC adapator protein and Capase-1 by ROS is an essential step in producing mature IL-1β and IL-18 from their precursor forms. mtROS can also oxidise mitochondrial DNA (mtDNA) to activate the NLR3 inflammasome.

### 6.5. Fibrous Cap Stability

Plaque rupture can lead to thrombus formation that occludes the vessel resulting in stroke and myocardial infarction. Plaque rupture results from degradation of the protective fibrous cap. Vulnerable plaques are characterized by a high ratio of macrophages to VSMCs, a large lipid-laden necrotic core and a thin fibrous cap [124]. Fibrous caps are formed by the deposition of collagen rich extracellular matrix components (ECM) and the accumulation of smooth muscle cells. 

Matrix metalloproteinases (MMP) are implicated in plaque rupture. The collagenase MMP subfamily, consisting of MMP-1, MMP-8 and MMP-13 are upregulated in atherosclerotic plaques and degrade ECM collagen [125]. The gelatinases MMP-2 and MMP-9 also cleave collagen and are upregulated in unstable plaques [126,127]. Consistent with MMPs promoting features of plaque vulnerability, macrophage expression of MMP-9 induces plaque disruption whilst MMP-12/ApoE double knock out mice have increased plaque VSMC content [128,129]. However, MMPs have heterogeneous effects and MMP-3/ApoE double knock out mice have increased plaque size, suggesting a protective role [129]. MMPs therefore have varying effects on plaque structure and development that may depend on disease stage.

MMP expression and activity is influenced by ROS. oxLDL upregulates MMP-1, MMP-2 and MMP-9 expression [130,131,132] whilst hydrogen peroxide activates MMP-1 and MMP-2 [133]. Monocyte NADPH oxidase-derived superoxide is associated with plasma levels of MMP-9, increases MMP-9 activity, and is associated with decreased plaque collagen content [134]. Vascular ROS may therefore affect plaque stability through effects on MMP expression and activity.

## 7. Antioxidant Therapies for Treating Atherosclerosis 

Given the widespread expression of pro-oxidant enzymes in the vasculature and pro-atherogenic nature of ROS, there is considerable interest in using antioxidant strategies to treat atherosclerosis. Antioxidants are compounds with high oxidising potential that can donate electrons to free radicals to neutralise them. Many proposed supplementary antioxidants are naturally occurring in fruits and vegetables so are consumed as part of a normal diet. However, it is unknown if the doses of vitamins consumed in food have any direct beneficial effects on vascular oxidative stress and cardiovascular disease [135]. 

While natural antioxidants have demonstrated potential in in vitro and in in vivo animal models, and even appear to be effective in short term human studies, no long term human study has yielded positive results to support antioxidant therapy in atherosclerosis [136]. Clinical trials investigating the use of dietary antioxidants such as vitamin E, beta carotene and higher doses of vitamin A to treat cardiovascular disease failed to demonstrate a protective effect [135]. Vitamin D supplementation also did not reduce the incidence of cardiovascular events in a large scale, randomised, placebo-controlled trial [137]. Smaller clinical trials with curcumin, the main polyphenol found in turmeric, have yielded initial promising results. Curcumin supplementation reduces LDL levels, increases NO production and reduces arterial stiffness [138]. However, due to a lack of adequately powered, long-term clinical trials it is unknown whether the promising antioxidant capacities of curcumin will work as an effective treatment in atherosclerosis.

Flavonoids have also been suggested as potential antioxidant-based therapies due to their ROS-scavenging and metal chelating properties [139]. Flavonoids are found in a wide variety of food sources such as cocoa, soy, tea and wine. A prospective cohort study of participants from the Danish Diet, Cancer and Health study revealed that a daily flavonoid intake of 1000 mg was associated with a 14% reduction in the risk of atherosclerotic cardiovascular disease [140]. Similar inverse relationships between flavonoid intake and cardiovascular disease risk have been reported in other prospective cohort studies [141]. However, a recent randomised controlled trial using cocoa extract supplementation failed to show a benefit on total cardiovascular events in older adults although there was a reduction in cardiovascular death [142]. Further studies would be required to clarify whether flavonoids will be of use in the treatment of atherosclerosis.

Resveratrol is a plant-derived polyphenol that has anti-oxidant properties. Resveratrol reduced the expression of inflammatory markers such as ICAM, VCAM and IL-8 in a double-blind, randomised control trial in healthy patients [143]. A recent multivariate analysis of 27 studies showed that long term administration of low doses of resveratrol (200–500 mg) was effective at lowering plasma triglycerides in obese and diabetic patients [144]. The higher levels of resveratrol were associated with reduced blood pressure and LDL, whilst the lower range showed the highest increase of HDL. The study therefore highlights that matching the dose and antioxidant treatment strategy to patients’ disease status may be required to improve outcomes. However, a clinical benefit of resveratrol in atherosclerosis has yet to be demonstrated.

Synthetic antioxidants also struggle to demonstrate beneficial clinical effects. The phenolic molecule succinobucol (AG-1067) has strong antioxidant and anti-inflammatory properties. However, it was not effective in the ARISE (Aggressive Reduction of Inflammation Stops Events) study which looked at the effects of succinobucol on cardiovascular disease. Succinobucol had no effect on the composite primary endpoint of time to first occurrence of cardiovascular death, resuscitated cardiac arrest, myocardial infarction, stroke, unstable angina, or coronary revascularisation [145]. Furthermore, the xanthine oxidase inhibitor febuxostat did not delay atherosclerosis progression in a multicentre, randomized controlled trial [146]. Inhibiting the release of signalling lipids from oxLDL with the lipoprotein-associated phospholipase A_2_ inhibitor darapladib has also failed to materialise as an effective treatment for atherosclerosis in two separate clinical trials [147,148]. Pharmacologically targeting vascular redox systems therefore remains a considerable challenge despite speculation about their therapeutic potential.

## 8. Future Directions 

A major limitation of many clinical studies investigating the use of antioxidants in atherosclerosis is that dosage is often not matched to the oxidative status of patients [149]. Trials that attempt to characterise the level of oxidative stress in patients and administer a corresponding level of antioxidant may provide greater insight into how vascular oxidative stress can be managed. This limitation extends to other confounding factors such as obesity status, age and sex which may require varying levels of antioxidants to see an effect on oxidative stress. 

The complexity of in vivo redox systems, both in humans and animal models, means that while studies investigating particular aspects of vascular redox status have yielded great insight, our understanding of the overall redox system is far from complete [150]. Many studies focus on measuring the effects of overexpressing or genetically deleting a particular source of ROS or antioxidant component. However, this often fails to capture the interplay between different redox systems and may lead to incorrect conclusions about the importance of a particular source of ROS. Future studies could try to account for this interplay and may so do by measuring other types of ROS and considering the activity of other enzymatic ROS sources [151]. 

Collectively the pre-clinical studies suggest that anti-oxidant therapy can protect endothelial function and reduce inflammation, features of plaque vulnerability and atherosclerosis burden. Optimising the targeting, timing and dose of anti-oxidants could therefore have significant clinical impact through reducing ischaemic symptoms, cardiovascular events, such as stroke and myocardial infarction, and cardiovascular mortality. We may also see beneficial effects on diseases beyond the cardiovascular system including chronic obstructive pulmonary disease, Alzheimer’s disease and cancer [152]. Given these potential therapeutic opportunities, advancing antioxidant therapy remains an important goal for future studies.

## 9. Conclusions

Oxidative stress is a highly complex component of atherosclerosis. ROS are required for vascular homeostasis yet are also important pathological molecules in disease progression. ROS are produced by a variety of enzymatic sources that are found in multiple cell types, which affords us a remarkable number of therapeutic targets. However, the universal expression of ROS-producing systems means that antioxidant-based therapies may need to be targeted to specific components of the vasculature to balance the redox status without compromising vascular health. Furthermore, the highly dynamic nature of the vascular redox status is not fully understood. Future mechanistic studies may need to take the interplay of ROS-producing enzymes and endogenous antioxidant systems into account and, despite promising pre-clinical data, establishing a clinical benefit for antioxidants in atherosclerosis remains a challenge.
